# Supplementary material for: Regional and global impact of CO2 uptake in the Benguela Upwelling System through preformed nutrients
Source: Nat Commun. 2023 May 4;14:2582. doi: 10.1038/s41467-023-38208-y (PMC10160069; doi:10.1038/s41467-023-38208-y)
Supplement: Supplementary file 1 — Supplementary Information [file 41467_2023_38208_MOESM1_ESM.pdf]

Supplementary Material

Regional and global impact of CO_2 uptake in the Benguela Upwelling System through preformed nutrients

Author Information

Claire Siddiqui*[1], Tim Rixen[1,2], Niko Lahajnar[2], Anja K. Van der Plas[3], Deon C. Louw[4], Tarron Lamont[5,6,7], Keshnee Pillay[5]

[1] Leibniz Centre for Tropical Marine Research - ZMT, Fahrenheitstrasse 6, 28359 Bremen, Germany

[2] Institute of Geology, Universität Hamburg, Bundesstrasse 55, 20146 Hamburg, Germany

[3] National Marine Information and Research Centre, PO Box 912, Swakopmund, 13001, Namibia

[4] Debmarine Namibia, 10 Dr Frans Indongo Street, Windhoek, 10005, Namibia

[5] Oceans & Coasts Research Branch, Department of Environment, Forestry and Fisheries, PO Box 52126, Victoria & Alfred Waterfront, Cape Town, 8000, South Africa

[6] Marine Research Institute & Department of Oceanography, University of Cape Town, Rondebosch, South Africa

[7] Bayworld Centre for Research & Education, 5 Riesling Road, Constantia, Cape Town, 7806 South Africa

Claire Siddiqui*: claire.siddiqui@leibniz-zmt.de

Figure S1

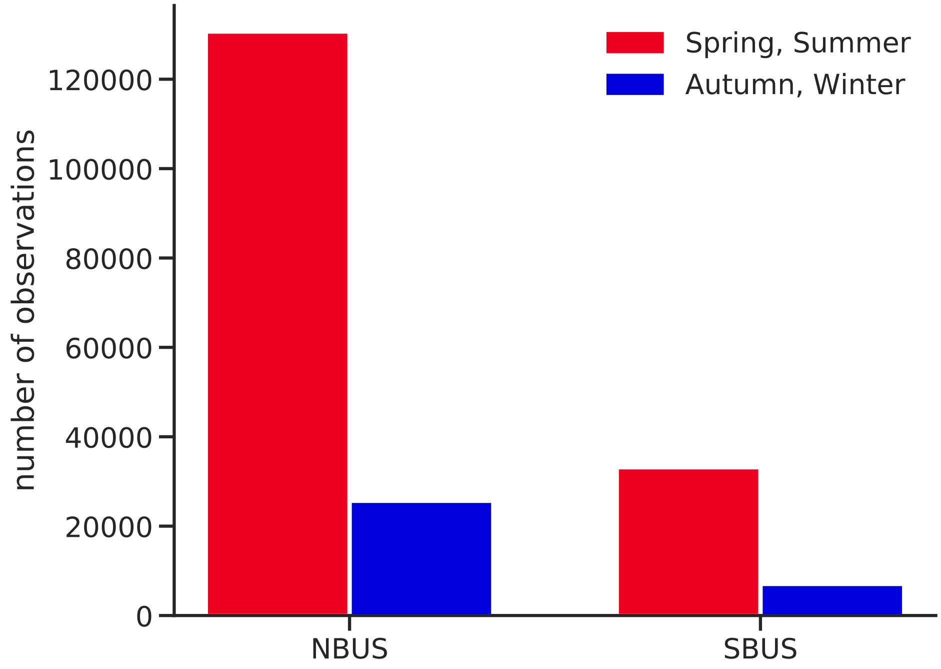

Figure S1. **Data coverage of sea surface pCO_2 measurements within the Benguela Upwelling System.** Number of observations on sea surface pCO_2 records per season within the northern (NBUS) and southern (SBUS) subsystem. During the spring/summer (Sept.-Mar., red) and autumn/winter season (Apr.-Aug., blue), observations amount to ~130,000 and ~25,000 in the NBUS, while the SBUS covers ~31,500 and ~6,000 measurements, respectively. The difference in number of pCO_2 records between the subsystems can partially be attributed to the defined upwelling area of the SBUS (177,600 km^2), which is ~53% smaller than the NBUS (377,400 km^2).

Figure S2

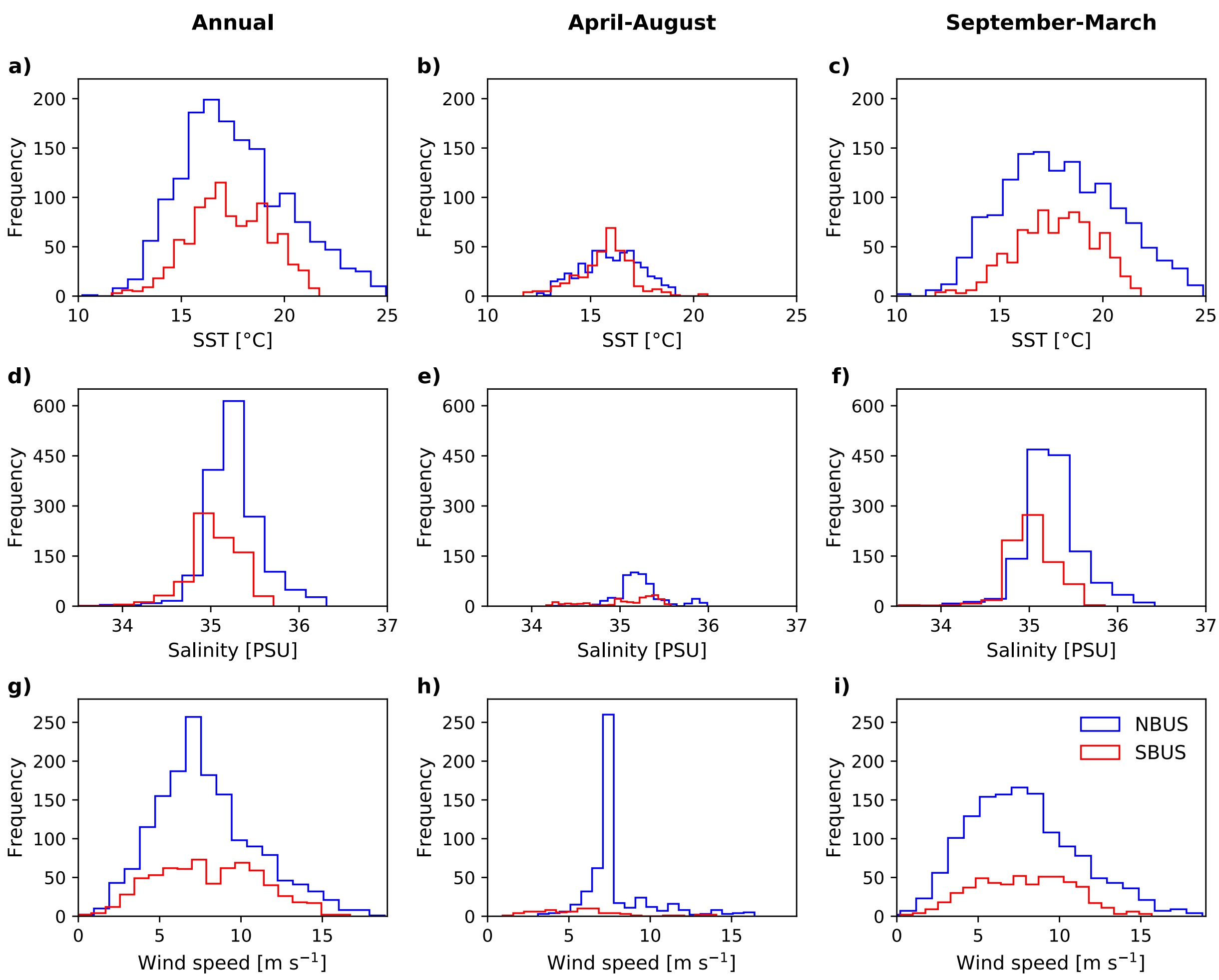

Figure S2. **Annual and seasonal histograms of individual parameters used for CO_2 flux calculations.** Histogram of Sea Surface Temperature (SST) **(a-c)**, salinity **(d-f)** and wind speed **(g-i)** within the northern (blue) and southern (red) Benguela Upwelling System (NBUS, SBUS) for different time periods, covering the austral spring and summer (September-March) and austral autumn and winter (April-August) seasons.

Figure S3

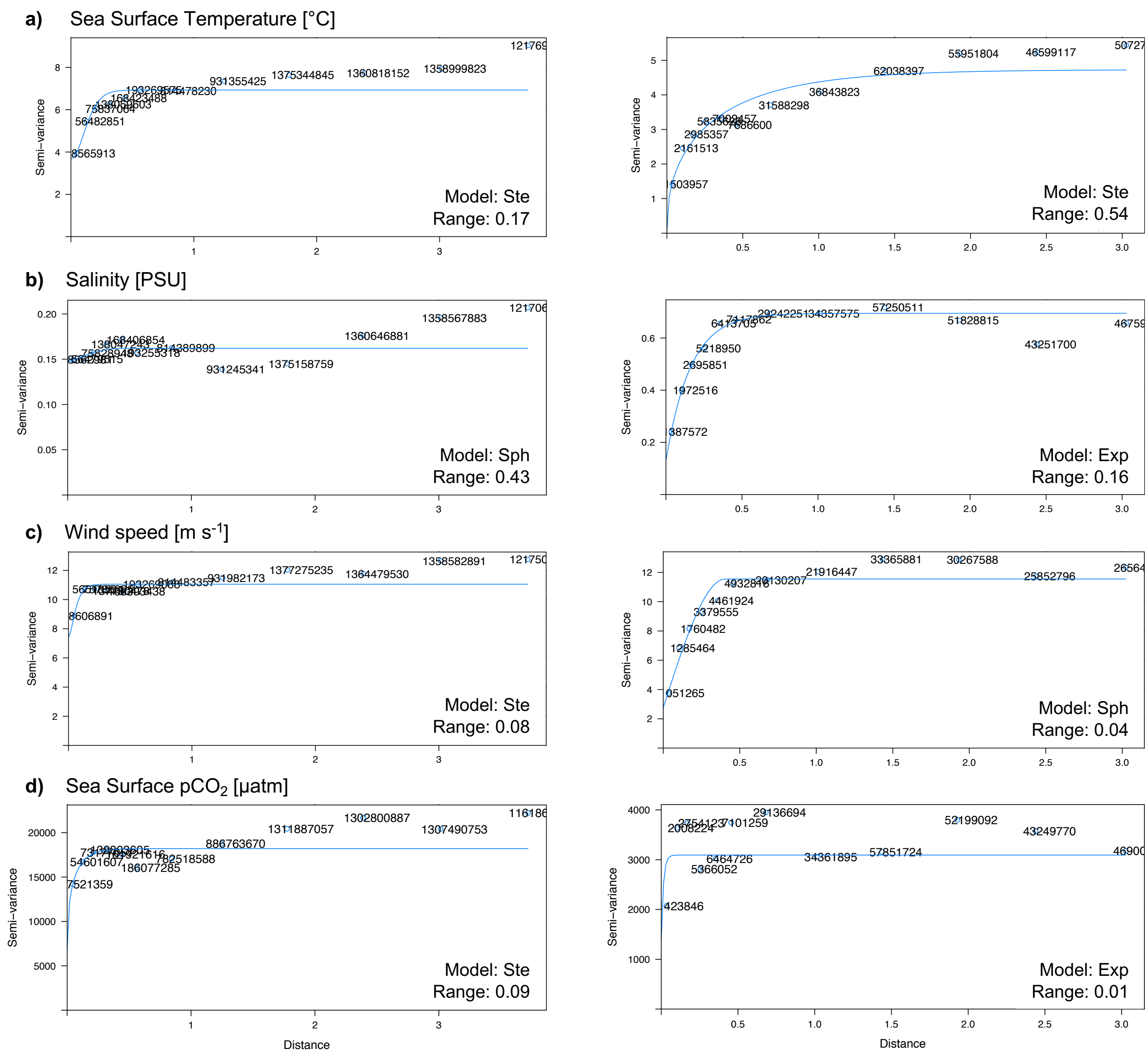

Figure S3. **Variogram models for ordinary kriging operation based on shipboard data. a** Sea Surface Temperature (SST), **b** salinity, **c** wind speed and **d** sea surface pCO_2 for the northern (left) and southern (right) Benguela Upwelling System used for the ordinary kriging interpolation. The blue line indicates the individual model fit of the variogram (Ste: Matern, M. Stein's parameterization; Sph: Spherical model; Exp: Exponential model)[1]. Numbers within each plot refer to the amount of paired measurements at each distance (here: in spherical distances), with range indicating the distance at which the spatial dependence (semi-variance, in parameter units) levels off.

Figure S4:

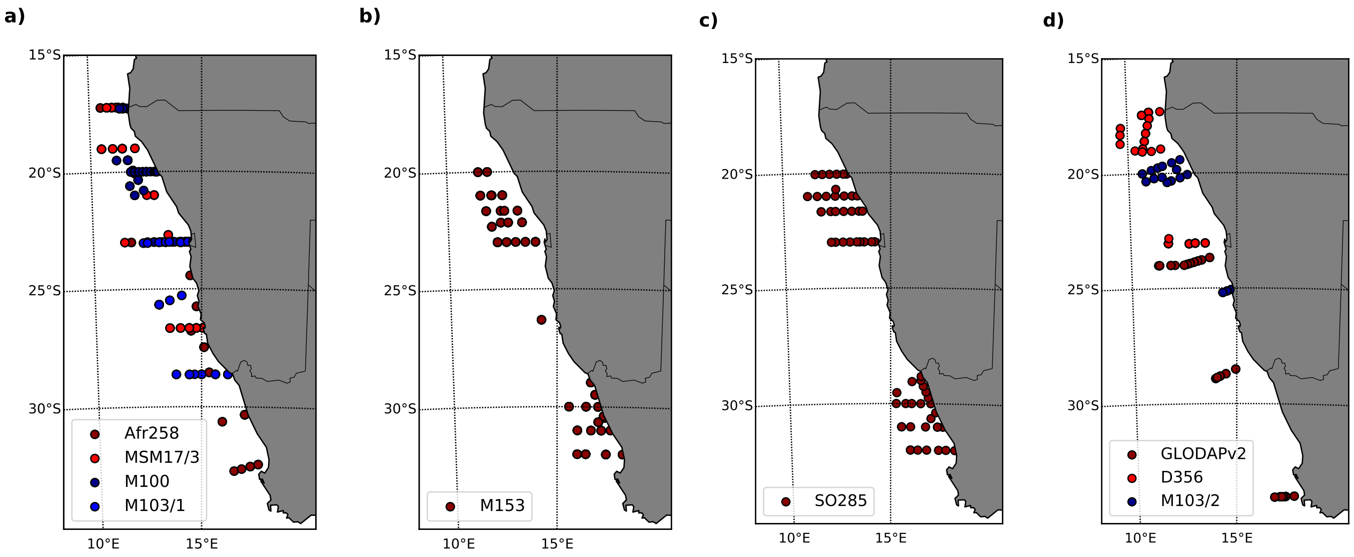

Figure S4. **Map of sampled stations within the Benugela Upwelling System.** Location of CTD/Rosette stations at which data collection of source water mass characteristics took place during the cruises **a** Africana 258 (Afr258), Maria S. Merian 17/3 (MSM17/3), Meteor 100 and 103/1 (M100, M103/1), **b** Meteor 153 (M153), **c** Sonne 285 (SO285) and **d** Discovery 356 (D356), Meteor 103/2 (M103/2), including Global Ocean Data Analysis Project (GLODAP) data (GLODAPv2). Maps were generated with python package matplotlib-basemap[2], and its dependencies PROJ-4[3] and GEOS[4], with country outlines sourced from the GSHHG Dataset[5].

Figure S5

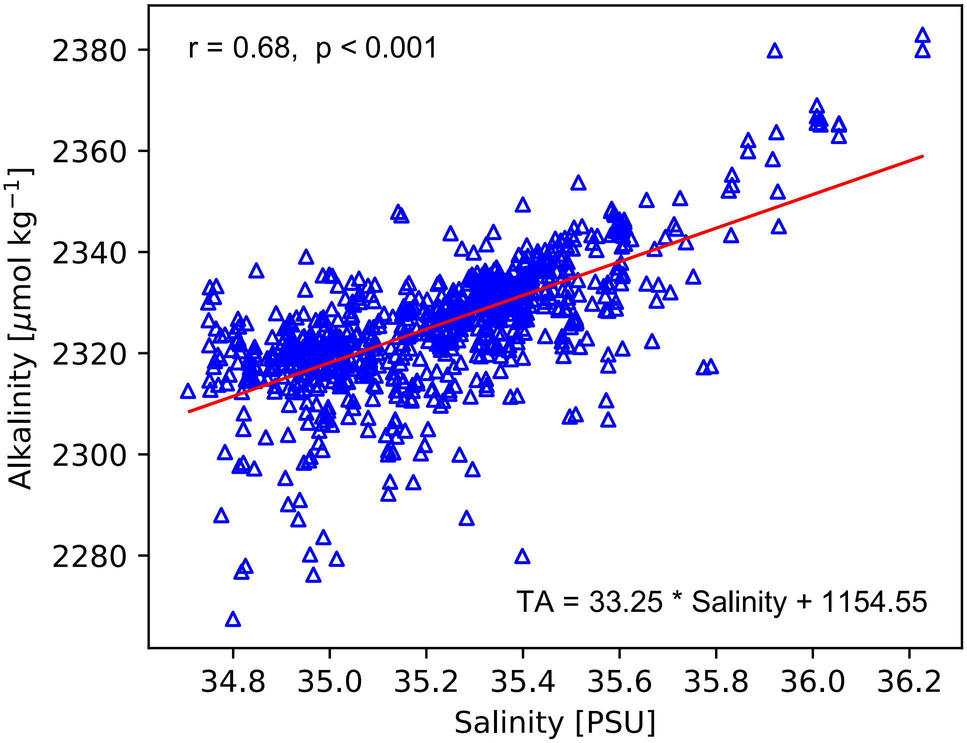

Figure S5. **Relation between sea surface alkalinity and salinity within the Benguela Upwelling System.** Linear regression based on sea surface alkalinity (TA, μmol kg^{-1}) and salinity (PSU) gathered from underway shipboard measurements during cruise SO285, and CTD surface profiles from all cruises (Supplementary Table 4) along the sampling locations (Supplementary Figure 4). Given are the linear regression function and line (in red), together with the Pearson's correlation coefficient and p-value.

Table S1. **List of expeditions to the Benguela Upwelling System with description of ID and timing of the cruises included in the pCO_2 database of this study.**

Research Vessel	Cruise ID	Time period
Maria S. Merian	MSM07/2	10/03/2008 – 20/03/2008
Meteor	M76/2	15/05/2008 – 05/06/2008
Africana	Afr258	02/12/2009 – 16/12/2009
Discovery	D356	10/09/2010 – 19/10/2010
Maria S. Merian	MSM17/3	20/01/2011 – 07/03/2011
Maria S. Merian	MSM18/4	24/07/2011 – 20/08/2011
Maria S. Merian	MSM19/1b	02/10/2011 – 11/10/2011
Meteor	M99	31/07/2013 – 23/08/2013
Meteor	M100/1	31/08/2013 – 01/10/2013
Meteor	M100/2	04/10/2013 – 21/10/2013
Meteor	M102	06/12/2013 – 23/12/2013
Meteor	M103/1	27/12/2013 – 18/01/2014
Meteor	M153	15/02/2019 – 31/03/2019

Table S2. **CO_2 exchange coefficients and flux rates for the northern and southern Benguela Upwelling System (NBUS, SBUS).**

Annual and seasonal mean air-sea CO_2 gas exchange rates, with positive flux values indicating CO_2 outgassing. Each CO_2 exchange coefficient represents the average value calculated on the basis of shipboard data gathered during the 14 cruises and those embedded into SOCAT v2020[6], which we interpolated on a 0.1°x0.1° grid using ordinary kriging. Uncertainties are given as the standard error. The corresponding *n* number of observations equals the number of effective grid cells outside of the spatial decorrelation lengths (except for *n**), which were derived for each parameter from semi variograms on the basis of observations during the respective time period (Annual, September-March, April-August).

**Parameter**	**NBUS**			**SBUS**		
	**Annual**	**Sep.-Mar.**	**Apr.-Aug.**	**Annual**	**Sep.-Mar.**	**Apr.-Aug.**
Sea Surface Temperature (°C)	17.63 ±1.97 *n*=1596	17.96 ±1.98 *n*=1391	15.99 ±0.31 *n*=477	17.34 ±1.27 *n*=951	17.70 ±1.24 *n*=686	15.67 ±0.91 *n*=225
Sea Surface Salinity (PSU)	35.26 ±0.39 *n*=1555	35.26 ±0.4 *n*=1382	35.23 ±0.12 *n*=512*	35.04 ±0.52 *n*=804	35.03 ±0.51 *n*=709	35.06± 0.28 *n*=233
Wind Speed (m/s)	7.88 ±2.98 *n*=1542	7.78 ±3.01 *n*=1391	7.84 ±0.95 *n*=512*	7.99 ±2.09 *n*=672	7.75 ±2.12 *n*=521	5.74 ±0.91 *n*=64
pCO_2,sw (µatm)	492.3 ±115,82 *n*=1578	481.0 ±116.82 *n*=1365	560.7 ±66.36 *n*=406	383.9 ±53.73 *n*=994	381.7 ±36.43 *n*=843	388.5 ±44.27 *n*=332
Solubility coefficient of CO_2, K_0	0.0346 ±0.0021	0.0342 ±0.002	0.0363 ±0.0004	0.0349 ±0.0013	0.0345 ±0.0013	0.0366 ±0.001
Piston velocity, *k*. upper / lower boundary	14.58 29.1 / 5.36	14.33 28.97 / 5.12	13.83 17.52 / 10.59	14.88 24.45 / 7.86	14.13 23.6 / 7.23	7.35 10.1 / 5.08
Carbon Flux rate (mol C m^{-2} yr^{-1}) upper / lower boundary	3.45 16.14 / -0.65	2.87 15.07 / -0.82	6.44 11.75 / 2.73	-1.38 1.68 / -2.11	-1.39 0.27 / -1.57	-0.61 0.58 / -1.57

Table S3. **Carbon flux rates of the northern and southern Benguela Upwelling System (NBUS, SBUS) based on various gas transfer coefficient (k) calculations (given in cursive numbers) with shipboard data on wind speeds that were interpolated using ordinary kriging.**

**Parameter**		**NBUS**			**SBUS**	
**(*k*, Flux rate*)**	**Annual**	**Sep.-Mar.**	**Apr.-Aug.**	**Annual**	**Sep.-Mar.**	**Apr.-Aug.**
Wanninkhoff, 1992	*24.92,*<br>5.89	*24.58,*<br>4.92	*23.53,*<br>10.96	*25,35,*<br>-2.36	*24.29,*<br>-2.39	*13.43,*<br>-1.11
Wanninkhoff & McGillis, 1999	*24.57,*<br>5.81	*23.81,*<br>4.77	*23.43,*<br>10.79	*25.48,*<br>-2.37	*23.43,*<br>-2.31	*9.01,*<br>-0.75
Nightingale et al., 2000	*15.35,*<br>3.63	*15.12,*<br>3.03	*14.57,*<br>6.79	*15.64,*<br>-1.45	*14.96,*<br>-1.47	*8.20,*<br>-0.68
McGillis et al., 2001	*14.99,*<br>3.55	*14.66,*<br>2.94	*14.19,*<br>6.61	*15.39,*<br>-1.43	*14.48,*<br>-1.42	*7.31,*<br>-0.61
Ho et al., 2006	*15.45,*<br>3.66	*15.19,*<br>3.05	*14.66,*<br>6.83	*15.78,*<br>-1.47	*15.02,*<br>-1.48	*7.79,*<br>-0.65
Wanninkhoff, 2014	*14.58,*<br>3.45	*14.33,*<br>2.87	*13.83,*<br>6.44	*14.88,*<br>-1.38	*14.17,*<br>-1.39	*7.35,*<br>-0.61

* Flux rate in mol C m^{-2} yr^{-1}

Table S4. **Shipboard data on source water mass characteristics gathered during various cruises to the northern and southern Benguela Upwelling System (NBUS, SBUS), including Global Ocean Data Analysis Project (GLODAP) data version 2.2020. Uncertainties are given as the standard error, with n corresponding to the number of observations.**

Cruise	**DIC**^{*1} (Dissolved Inorganic Carbon)	**TA**^{*1} (Total Alkalinity)	$\mathbf{O_2}$ ^{*1} (Oxygen)	$\mathbf{P_{total}}$ ^{*1} (Total Phosphate)	$\mathbf{P_{Pref}}$ ^{*1} (Preformed Phosphate)	$\mathbf{P_{Pref}}$ **-%** (%-Preformed Phosphate)	$\mathbf{N_{total}}$ ^{*1} (Total Nitrate)	$\mathbf{N_{Pref}}$ ^{*1} (Preformed Nitrate)	$\mathbf{N_{Pref}}$ **-%** (%-Preformed Nitrate)	**SWT**^{*2} (Source Water Temperature)	**SWS**^{*3} (Source Water Salinity)
**NBUS**											
Afr258	2200.32 ±9.44<br>n=26	2301.26 ±1.9<br>n=48	47.34 ±5.95<br>n=49	2.07 ±0.06<br>n=48	0.51 ±0.04<br>n=47	24.91 ±1.6<br>n=47	27.86 ±0.8<br>n=48	5.59 ±0.6<br>n=39	19.26 ±1.9<br>n=39	11.8 ±0.19<br>n=49	35.09 ±0.02<br>n=49
D356	2220.40 ±5.03<br>n=82	2306.02 ±1.05<br>n=90	44.36 ±6.43<br>n=32	1.88 ±0.04<br>n=97	0.29 ±0.03<br>n=29	16.39 ±1.3<br>n=29	32.62 ±0.8<br>n=97	9.01 ±0.3<br>n=29	27.94 ±1.3<br>n=29	11.94 ±0.12<br>n=102	35.12 ±0.02<br>n=102
MSM17/3	2232.95 ±5.61<br>n=47	2304.76 ±1.42<br>n=45	58.69 ±8.69<br>n=47	2.33 ±0.12<br>n=45	0.86 ±0.09<br>n=45	34.3 ±2.4<br>n=45	27.12 ±0.8<br>n=44	5.77 ±0.6<br>n=35	21.76 ±2.2<br>n=35	11.65 ±0.21<br>n=48	35.09 ±0.03<br>n=48
M100	-	2308.20 ±2.59<br>n=118	85.33 ±47<br>n=134	1.84 ±0.04<br>n=130	0.54 ±0.03<br>n=129	29.64 ±2.0<br>n=129	25.99 ±0.4<br>n=130	8.76 ±0.4<br>n=107	32.91 ±1.8<br>n=107	11.9 ±0.1<br>n=134	35.07 ±0.01<br>n=134
M103/1	-	2302.50 ±1.45<br>n=41	67.37 ±9.44<br>n=51	1.90 ±0.12<br>n=33	0.72 ±0.06<br>n=33	37.54 ±1.4<br>n=33	22.68 ±1.1<br>n=32	5.99 ±0.5<br>n=28	26.26 ±1.7<br>n=28	11.37 ±0.19<br>n=51	35.01 ±0.03<br>n=51
M103/2	-	2306.57 ±2.9<br>n=16	35.68 ±4.49<br>n=53	2.25 ±0.05<br>n=49	0.61 ±0.03<br>n=49	26.59 ±1.0<br>n=49	25.03 ±1.3<br>n=49	4.59 ±0.3<br>n=33	14.64 ±0.9<br>n=33	11.49 ±0.19<br>n=53	35.05 ±0.02<br>n=53
M153	2314.23 ±6.9<br>n=95	2298.73 ±1.7<br>n=97	85.44 ±2.8<br>n=267	1.87 ±0.03<br>n=96	0.54 ±0.01<br>n=96	29.29 ±0.1<br>n=96	24.52 ±0.5<br>n=96	4.58 ±0.4<br>n=96	18.06 ±1.7<br>n=96	11.5 ±0.09<br>n=267	35.04 ±0.01<br>n=267
SO285	2250,51 ±2.82<br>n=134	2308.53 ±0.59<br>n=140	75.05 ±2.69<br>n=287	-	-	-	-	-	-	11.8 ±0.07<br>n=287	35.06 ±0.01<br>n=287
GLODAP	2209.40 ±5.41<br>n=39	2298.56 ±082<br>n=39	115.32 ±7.22<br>n=64	1.73 ±0.05<br>n=61	0.58 ±0.02<br>=61	34.47 ±1.3<br>n=61	26.09 ±0.8<br>n=62	7.27 ±0.4<br>n=62	29.03 ±1.5<br>n=62	10.77 ±0.19<br>n=64	34.93 ±0.02<br>n=64
mean	2237.97	2303.9	67.77	1.97	0.59	29.27	26.49	6.44	23.73	11.58	35.05
std. error	18.48	1.35	8.47	0.08	0.06	2.50	1.04	0.61	2.22	0.13	0.02
**SBUS**											
Afr258	2165.47 ±9.78<br>n=34	2299.53 ±1.81<br>n=34	131.94 ±10.23<br>n=39	1.89 ±0.11<br>n=35	0.99 ±0.08<br>n=35	52.21 ±2.3<br>n=35	21.42 ±0.8<br>n=35	7.43 ±0.4<br>n=34	37.35 ±2.4<br>n=34	10.45 ±0.18<br>n=39	34.87 ±0.02<br>n=39
M103/1	-	2302.14 ±1.79<br>n=20	138.26 ±10.86<br>n=19	1.67 ±0.11<br>n=20	0.72 ±0.04<br>n=19	44.15 ±1.9<br>n=19	21.35 ±1.1<br>n=20	6.47±0.6<br>n=19	31.89 ±3.1<br>n=19	10.42 ±0.25<br>n=20	34.84 ±0.03<br>n=20
M153	2246.17 ±6.2<br>n=102	2283.94 ±2.6<br>n=101	146.87 ±3.71<br>n=245	1.6 ±0.05<br>n=79	0.75 ±0.02<br>n=79	49.78 ±0.2<br>n=79	20.57 ±0.7<br>n=79	7.67 ±0.5<br>n=79	40.52 ±2.7<br>n=79	10.5 ±0.06<br>n=265	34.83 ±0.01<br>n=265
SO285	2203.06 ±4.03<br>n=150	2303 ±0.51<br>n=154	156.39 ±2.18<br>n=391	-	-	-	-	-	-	10.09 ±0.07<br>n=391	34.8 ±0.01<br>n=391
GLODAP	2160.73 ±4.38<br>n=52	2297.98 ±0.79<br>n=51	180.81 ±4.75<br>n=52	1.48 ±0.05<br>n=63	0.75 ±0.02<br>n=63	52.14 ±0.98<br>n=63	21.02 ±0.9<br>n=47	8.32 ±0.2<br>n=46	41.37 ±1.4<br>n=46	10.19 ±0.15<br>n=105	34.83 ±0.02<br>n=105
mean	2193.86	2297.49	150.74	1.66	0.80	48.85	21.06	7.44	37.73	10.33	34.83
std. error	22.91	3.89	9.57	0.09	0.06	2.00	0.20	0.36	2.13	0.09	0.01

Units in ^{*1} µmol kg^{-1}, ^{*2} °C, ^{*3} PSU

Table S5. **List of accession codes to all files included in the PANGAEA database on sea surface and water column characteristics as used in this study.**

Research Vessel	Cruise ID	Accession Code on PANGAEA	
		Sea Surface pCO_2	Water column characteristics
Maria S. Merian	MSM07/2	https://doi.pangaea.de/10.1594/PANGAEA.880384	-
Meteor	M76/2	https://doi.pangaea.de/10.1594/PANGAEA.880395	-
Africana	Afr258	https://doi.pangaea.de/10.1594/PANGAEA.880382	https://doi.pangaea.de/10.1594/PANGAEA.779586
Discovery	D356	https://doi.pangaea.de/10.1594/PANGAEA.880383	https://doi.pangaea.de/10.1594/PANGAEA.779634
Maria S. Merian	MSM17/3	https://doi.pangaea.de/10.1594/PANGAEA.880385	https://doi.pangaea.de/10.1594/PANGAEA.779638
Maria S. Merian	MSM18/4	https://doi.pangaea.de/10.1594/PANGAEA.880392	-
Maria S. Merian	MSM19/1b	https://doi.pangaea.de/10.1594/PANGAEA.880393	-
Meteor	M99	https://doi.pangaea.de/10.1594/PANGAEA.957810	-
Meteor	M100/1	https://doi.pangaea.de/10.1594/PANGAEA.957810	https://doi.pangaea.de/10.1594/PANGAEA.853623
Meteor	M100/2	https://doi.pangaea.de/10.1594/PANGAEA.957810	https://doi.pangaea.de/10.1594/PANGAEA.853623
Meteor	M102	https://doi.pangaea.de/10.1594/PANGAEA.957810	-
Meteor	M103/1	https://doi.pangaea.de/10.1594/PANGAEA.957810	https://doi.pangaea.de/10.1594/PANGAEA.853624
Meteor	M153	https://doi.pangaea.de/10.1594/PANGAEA.957810	https://doi.pangaea.de/10.1594/PANGAEA.957862
Sonne	SO285	https://doi.pangaea.de/10.1594/PANGAEA.957810	https://doi.pangaea.de/10.1594/PANGAEA.957862

Supplementary References

1 Pebesma, E. Multivariable geostatistics in S: The GSTAT package. *Computers & Geosciences* **30**, 683-691, doi:10.1016/j.cageo.2004.03.012 (2004).

2 Hunter, J. D. Matplotlib: A 2D graphics environment. *Computing in Science & Engineering* **9**, 90-95 (2007).

3 PROJ contributors. PROJ coordinate transformation software library. Open Source Geospatial Foundation. doi:10.5281/zenodo.5884394. URL https://proj.org/ (2023).

4 GEOS contributors. GEOS computational geometry library. Open Source Geospatial Foundation. URL https://libgeos.org/ (2021).

5 Wessel, P. & Smith, W. H. F. A Global Self-consistent, Hierarchical, High-resolution Shoreline Database. *Journal of Geophysical Research* **101**, 8741-8743 (1996).

6 Bakker, D. C. E. *et al.* A multi-decade record of high-quality fCO2 data in version 3 of the Surface Ocean CO2 Atlas (SOCAT). *Earth Syst. Sci. Data* **8**, 383-413 (2016).
